# Analysis of Virulence Genes Among Methicillin Resistant *Staphylococcus aureus* (MRSA) Strains

**DOI:** 10.5812/jjm.10741

**Published:** 2014-06-01

**Authors:** Seyedeh Mahsan Hoseini Alfatemi, Mohammad Motamedifar, Nahal Hadi, Hadi Sedigh Ebrahim Saraie

**Affiliations:** 1 Department of Bacteriology and Virology, Shiraz Medical School, Shiraz University of Medical Sciences, Shiraz, IR Iran; 2 Shiraz HIV/Aids Research Center (SHARC), Department of Bacteriology and Virology, Medical School, Shiraz University of Medical science, Shiraz, IR Iran

**Keywords:** Methicillin-resistant *Staphylococcus aureus*, Iran

## Abstract

**Background:**

*Staphylococcus aureus* is amongst major human pathogens both in hospitals and the community. This bacterium is an opportunistic pathogen responsible for a large number of self-limiting and even life-threatening diseases in humans. Methicillin resistant *S. aureus* (MRSA) strains are common causes of emerging nosocomial infections and are considered as a major problem for public health.

**Objectives:**

We aimed to study the profile of some virulence genes including: *sea, seb, sed, tst, eta, etb, LuKS/F-PV, hla and hld* in methicillin-resistant *S. aureus* by the PCR technique.

**Materials and Methods:**

A total of 345 isolates of *S. aureus* were collected from clinical specimens of patients referred to teaching hospitals of Shiraz; identification was done by biochemical (catalase, coagulase and DNase) and molecular tests. One hundred and forty six isolates of methicillin-resistant *S. aureus* (MRSA) were obtained and the presence of some toxin genes in these isolates was investigated by the polymerase chain reaction (PCR) technique.

**Results:**

The results showed that among the 345 isolates of *S. aureus*, 148 were confirmed as MRSA by screening with the cefoxitin disc diffusion (30 µg) method. Also among the 148 MRSA isolates, 146 isolates were confirmed as methicillin-resistant by molecular methods. The results showed that the frequency of methicillin-resistant and methicillin-sensitive *S. aureus* isolates during 2012 to 2013 in Namazi and Faghihi hospitals were 146 (42.3%) and 199 (57.7%), respectively. Besides, among the 146 confirmed MRSA isolates, 36.98% (54 isolates) and 63.02% (92 isolates) were related to female and male, respectively. The largest number of cases belonged to sputum samples (58 out of 146). The frequency of the *eta, etb, sed, LuKS/F-PV, seb, tst, sea, hld* and *hla* genes were 0.68%, 2.05%, 2.05%, 5.47%, 10.95%, 11.64%, 27.39%, 84.24% and 93.15%, respectively. In addition, amongst all examined genes, *hla* (93.15%) and *eta* (0.68%) genes had the highest and lowest frequencies, respectively. The greatest coexistence of genes was observed for the *hla + hld* gene combination (48.83%). The results of our study indicate that 98.63% of the isolates were positive for at least one of the virulence genes.

**Conclusions:**

The relative higher frequency of some virulence genes in this study may reflect the emergence of isolates containing these genes in Shiraz medical centers.

## 1. Background

*Staphylococcus aureus* is a Gram-positive bacterium found in axillae, nose, groin, perineal area (males), mucous membranes, mouth, mammary glands, hair, and intestinal, genitourinary and upper respiratory tracts of human and sometimes leads to production of pus and abscesses, sepsis and even fatal septicemia (1-3). *S. aureus* is amongst major human pathogens both in hospitals and the community (4). The bacterium is an opportunistic pathogen that can bring about a multiplicity of self-limiting and even life-threatening di*sea*ses in humans (2). Antibiotics such as cephalexin and cloxacillin are usually used to treat staphylococci infections (5). Many strains of *S. aureus* have increased resistance to multiple different classes of antibiotics (6). Methicillin resistant *S. aureus* (MRSA) strains are common causes of nosocomial infections (7). Increasing resistance to vancomycin, which is administered intravenously and used to treat MRSA, has been documented in many hospitals (6-8).

The universal emerge of MRSA is considered as a major problem for public health (9, 10). The ability of *S. aureus* strains to cause disease depends on a wide range of virulence factors that contribute to colonization and disease in the host (10). *S. aureus* produces an extracellular protein with low molecular weight toxins and virulence factors. Several types of staphylococcal enterotoxins have been reported, including: A, B, C, D, E, F, G, H, I, G, K, L, M, N, O, P, Q and R and their associated genes are: *sea*, *seb*, *sec*, *sed*, *see*, *seg*, *seh*, *sei*, *sej*, *sek*, *sel*, *sem*, *sen*, *seo*, *sep*, *seq* and *ser*, respectively. The enterotoxins are similar to each other in terms of structure and biological activity, but they are different as far as antigen characteristics are concerned (11). More than 90% of staphylococcal enterotoxin food poisonings are related to the A-E groups. The A and D enterotoxins are the major cause of staphylococcal food poisoning (11).

Staphylococcal enterotoxins are heat resistant and if are present in dairy products such as milk and cheese, raw vegetables and candies, they could lead to food poisoning. Staphylococcal enterotoxin poisoning in human causes nausea, vomiting and occasionally leads to diarrhea and muscular and abdominal pain (12). Some *S. aureus* strains produce various types of staphylococcal enterotoxins (SEs), toxic shock syndrome toxin 1 (TSST-1), exfoliative toxins (ETs), hemolysis (alpha, beta, gamma and delta) and Panton Valentine leukocidin (PVL). Toxic shock syndrome toxin 1 and ETS toxins cause toxic shock syndrome and staphylococcal peeling skin syndrome, respectively (13). The alpha, beta, delta, gamma hemolysin and leukocidin toxins are coded by *hla*, *hlb*, *hld*, *hlg* and *lukD-lukE* genes, respectively. These toxins are important in creating a large number of staphylococcal infections (14). Panton Valentine leukocidin toxin has been reported to be associated with severe illnesses in children and adolescents without a prior visit to health care institutions (15).

## 2. Objectives

In this study, we aimed to investigate the profile of some virulence genes including: *sea*, *seb*, *sed*, *tst*, *eta*, *etb*, *LuKS/F-PV*, *hla* and *hld* in methicillin-resistant *S. aureus* isolates by using the polymerase chain reaction (PCR) technique at two major teaching hospitals in Shiraz, Iran. This study is important for detection of the profile of some virulence genes involved in toxicity of the MRSA pathogenic isolates. Besides, the information obtained from the prevalence of toxin genes in MRSA strains can greatly assist the health authorities for prevention, control and treatment of the related di*sea*ses.

## 3. Materials and Methods

### 3.1. Bacterial Strains and Culture Media

In this study, bacteria were isolated from patients of two teaching hospitals (Namazi and Faghihi) in Shiraz (Iran) from 2012 to 2013. Samples from various clinical specimens including blood, pus, wound, urine were transferred to the laboratory and subjected to diagnostic tests, such as catalase test, culturing on Mannitol Salt Agar, coagulase tube test and DNase.

### 3.2. Determination of MRSA Strains and Antibiotic Susceptibility

For differentiation of MRSA from methicillin-sensitive *S. aureus* (MSSA) strains, Muller Hinton Agar medium (Oxoid Ltd, UK) was used. The strains in a liquid medium of 0.5 Mc Farland standard concentration were grown in Muller Hinton Agar medium and 30 µg cefoxitin antibiotic discs (Mast Group Ltd, UK) were placed on the medium and incubated for 18 hours at 37°C (16). Next, the diameter of the clear zone around the discs was measured by standards of Clinical Laboratory Standard Institute (CLSI) and the types of MRSA or MSSA strains were identified (Inhibition zone diameter ≤ 21 indicated MRSA and inhibition zone diameter ≥ 22 indicated MSSA) (17).

### 3.3. DNA Extraction

DNA was extracted using the phenol-chloroform method (18). DNA samples were dissolved in trisacetate-Ethylenediaminetetraacetic acid (Tris-EDTA) buffer (HCl 10 mM Tris, 1 mM EDTA, pH = 7.4), and DNA concentration was determined by spectrophotometer at A260 based on µg/ml concentration. In this study, the quantity of DNA samples used ranged from 10 to 1000 ng. DNA obtained was preserved at -20°C.

### 3.4. Polymerase chain reaction Assay and Electrophoresis for Detection of Toxin Genes

All MRSA isolates were assayed for the presence of the *sea*, *seb*, *sed*, *tst*, *eta*, *etb*, *LuKS/F-PV*, *hla*, *hld* and *mecA* genes by polymerase chain reaction (PCR) using previously described primers (19, 20) (Table 1). In the present study, the standard strains u*sed* for each toxin gene and MRSA are summarized in (Table 2); distilled water was u*sed* as the negative control. For PCR, forward and reverse primers (Table 3) were diluted to reach a concentration of 100 pM. After preparing the PCR mix (Tables 4,5), amplifications were performed following programs stated in Table 6. PCR products were mixed with 1 µL of loading buffer solution and carefully loaded in the wells of the agarose gel (1.5%) and electrophoresed at 75 V for 90 minutes. The gel was then stained with ethidium bromide (Merck, Germany) solution for 15 minutes and observed under a UV trans-illuminator.

**Table 1.** The Oligonucleotide Sequences and Amplicon Size of Each Gene Used in This Study (19, 20)

	Oligonucleotide Sequence (5’-3’)	Amplicon Size, bp
***sea***		102
GSEAR-1	GGTTATCAATGTGCGGGTGG	
GSEAR-2	CGGCACTTTTTTCTCTTCGG	
***seb***		164
GSEBR-1	GTATGGTGGTGTAACTGAGC	
GSEBR-2	CCAAATAGTGACGAGTTAGG	
***sed***		278
GSEDR-1	CCAATAATAGGAGAAAATAAAAG	
GSEDR-2	ATTGGTATTTTTTTTCGTTC	
***tst***		326
GTSSTR-1	ACCCCTGTTCCCTTATCATC	
GTSSTR-2	TTTTCAGTATTTGTAACGCC	
***eta***		93
GETAR-1	GCAGGTGTTGATTTAGCATT	
GETAR-2	AGATGTCCCTATTTTTGCTG	
***etb***		226
GETBR-1	ACAAGCAAAAGAATACAGCG	
GETBR-2	GTTTTTGGCTGCTTCTCTTG	
***LukS/F-PV***		443
PVL-1	ATCATTAGGTAAAATGTCTGGACATGATCCA	
NPVL-2	GCATCAAGTGTATTGGATAGCAAAAGC	
***hla***		209
HLA-1	CTGATTACTATCCAAGAAATTCGATTG	
HLA-2	CTTTCCAGCCTACTTTTTTATCAGT	
***hld***		111
HLD-1	AAGAATTTTTATCTTAATTAAGGAAGGAGTG	
HLD-2	TTAGTGAATTTGTTCACTGTGTCGA	
***mecA***		147
MECA-1	GTGAAGATATACCAAGTGATT	
MECA-2	ATGCGCTATAGATTGAAAGGAT	

**Table 2.** The Control Strains Used in this Study for Each Toxin Gene of *S. aureus*

Strain Names	Target Genes
***S. aureus*** ** COL**	*seb*
***S. aureus*** ** ATCC14458**	MRSA, *LukS/F-PV*
***S. aureus*** ** N315**	*hla*, *hld*, *eta*
***S. aureus*** ** JCSC/4469**	*etb*, *sed*, *tst*, *sea*

**Table 3.** The Amount of Injected Water for Dilution of Primers to a Concentration of 100 pM

Primer Name	µL of Water
**GSEAR-1**	166.03
**GSEAR-2**	367.59
**GSEBR-1**	190.23
**GSEBR-2**	200.96
**GSEDR-1**	270.46
**GSEDR-2**	535.28
**GTSSTR-1**	174.45
**GTSSTR-2**	346.17
**GETAR-1**	359.54
**GETAR-2**	369.59
**GETBR-1**	192.28
**GETBR-2**	340.52
**PVL-1**	155.89
**PVL-2**	234.71
**HLA-1**	219.50
**HLA-2**	181.48
**HLD-1**	240.84
**HLD-2**	262.45
**MECA-1**	304.94
**MECA-2**	197.89

**Table 4.** Reagents Used for PCR of All Genes Except for *LukS/F-PV* Gene

Materials	Volume Used, µL	Concentrations
**dNTPs (Cinna Gen, Iran)**	1	200 µm
**MgCl**_**2**_ ** (Merck, Germany)**	1.5	1.5 mM
**PCR buffer (Cinna Gen, Iran)**	2.5	1 X
**Forward primer (Cinna Gen, Iran)**	1	10 pmol/µL
**Reverse primer (Cinna Gen, Iran)**	1	10 pmol/µL
**Taq polymerase (Cinna Gen, Iran)**	0.25	1 unit
**Double-distilled water (Cinna Gen, Iran)**	14.75	-
**DNA template**	3	-
**-**	final volume = 25	-

**Table 5.** Reagents Used for PCR of *LukS/F-PV* Gene

Materials (Co. Name, Country)	Volume Used, µL	Concentration
**dNTPs(Cinna Gen, Iran)**	1.5	200 µM
**MgCl**_**2**_ ** (Merck, Germany)**	1	1.5 mM
**PCR buffer (Cinna Gen, Iran)**	2.5	1 X
**Forward primer (Cinna Gen, Iran)**	1	10 pmol/µL
**Reverse primer (Cinna Gen, Iran)**	1	10 pmol/µL
**Taq polymerase (Cinna Gen, Iran)**	0.5	1 unit
**Double-distilled water (DDW)**	15.5	-
**DNA template**	2	-
**-**	final volume = 25	-

**Table 6.** Thermal Cycler Programs Used in This Study

Cycle Number	Time	Temperature, °C	Definite	Steps	Programs
**1**	5 min	94	Initial denaturation	1	program 1
**30**	30 sec	95	Denaturation	1	program 2
**30**	45 sec	50-60 ^a^	Annealing	2	program 2
**30**	1 min	72	Extension	3	program 2
**1**	7 min	72	final extension	1	program 3

^a^ Change in range is inserted suit to different genes. Annealing temperature for the genes were followed as: *eta* and *etb* (54°C); *tst*, *mecA*, *sed* and *seb* (50°C); *LukS/F-PV* and *sea* (60°C); *hla* and *hl*d (58°C).

### 3.5. Statistical Analysis

For investigating the relationship between data obtained at different stages of this study, data were analyzed in statistical package for social sciences (SPSS) software, version 11.5. Chi-square test was used to determine the relationship between the variables with P < 0.05 considered statistically significant.

## 4. Results

### 4.1. Biochemical Characterization of Methicillin-Resistant S. aureus Isolates

**Figure 1.**
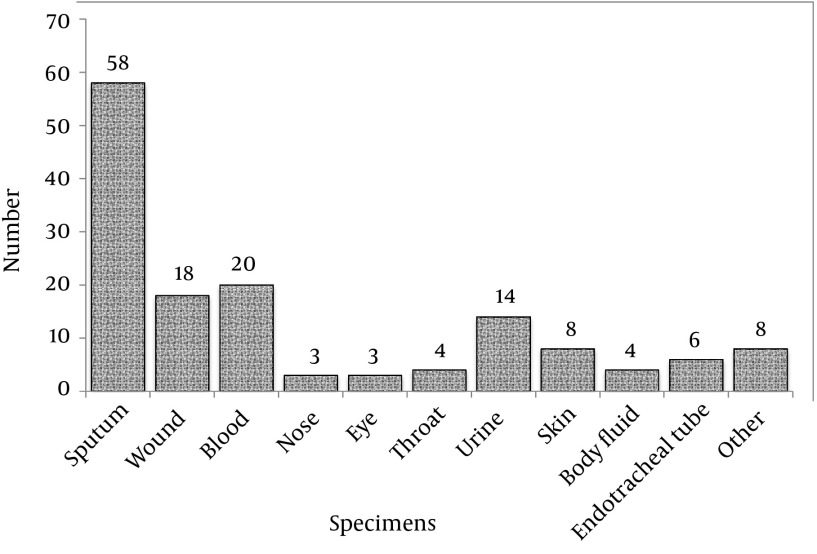
The Frequency of Isolates Collected From Different Sources of Infection

From the samples, various clinical specimens were subjected to diagnostic tests and in total, 345 isolates were identified as *S. aureus*. Among the 345 isolates of *S. aureus*, 148 were confirmed as MRSA by screening with the cefoxitin disc (30 µg) diffusion method. Using molecular methods, 146 methicillin-resistant isolates were confirmed to have the *mecA* gene using the PCR method, as described earlier. The prevalence rates of MRSA and MSSA isolates were 146 (42.3%) and 199 (57.7%), respectively. From the 146 confirmed isolates, 77 (52.7%) and 69 (47.3%) MRSA belonged to Faghihi hospital and Namazi hospital, respectively. However, the results indicated that the numbers of MRSA isolates obtained from the two hospitals were not significantly different (P < 0.05). The rate of MRSA isolation from females and males was 54 (36.98%) and 92 (63.02%), respectively. The frequencies of isolates collected from different sources of infection are shown in (Figure 1). The results indicated that among the 146 isolates, sputum, blood and urine specimens with 58, 20 and 14 cases, respectively, had the greatest proportions. The results also showed that the nose and eye specimens had the lowest proportion with only three cases (Figure 1).

### 4.2. Result of Molecular Tests

**Figure 2.**
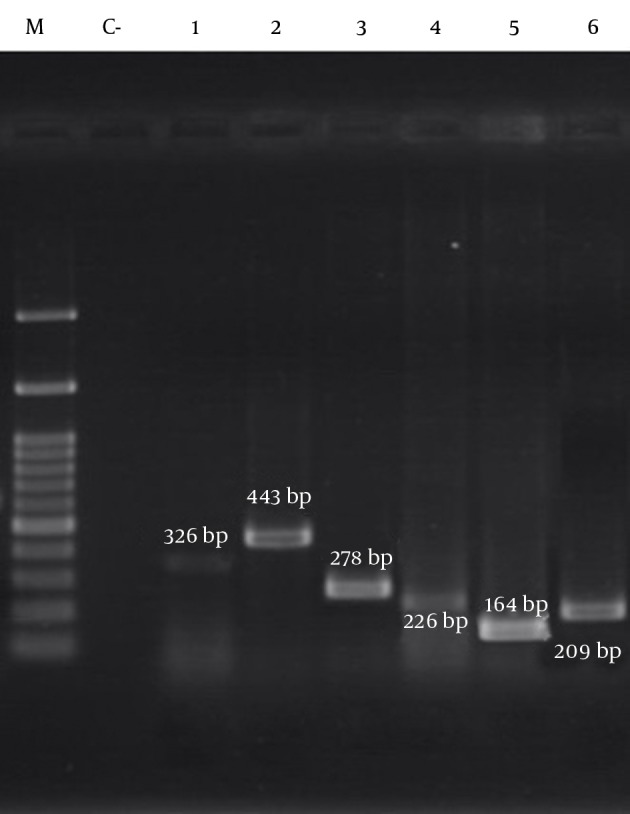
Patterns of Agarose Gel Electrophoresis Showing PCR Amplification Products for the Isolated *S. aureus* Genes. Lanes M, DNA molecular size marker (100-bp ladder; Cinna Gen, Iran); C-: negative control; lane 1: *tst*; lane 2: LukS /F-PV; lane 3: *sed*; lane 4: *etb*; lane 5: *seb*; lane 6: *hla*

**Figure 3.**
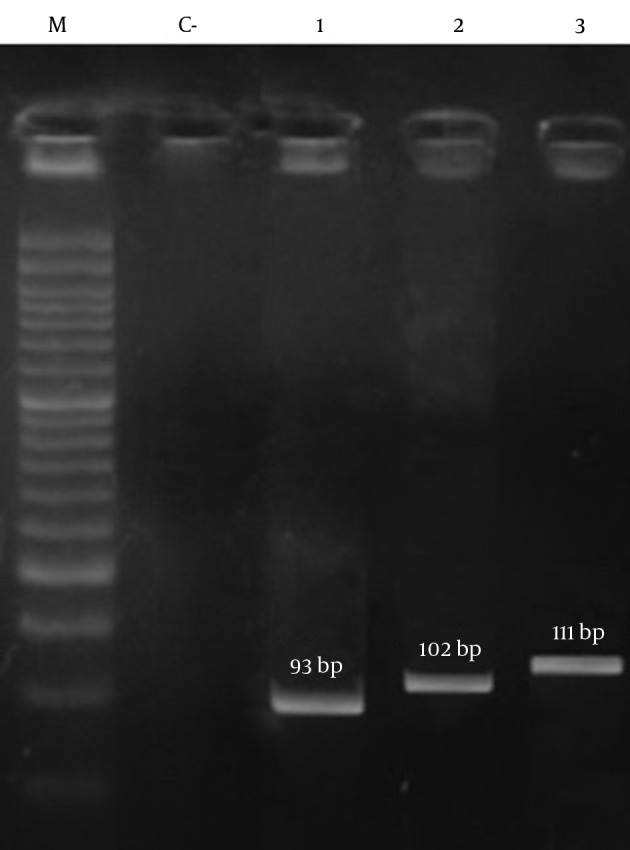
Patterns of Agarose Gel Electrophoresis Showing PCR Amplification Products for the Isolated *S. aureus* Genes. Lanes M, DNA molecular size marker (50-bp ladder; Cinna Gen, Iran); C-: negative control; lane 1: *eta*; lane 2: *sea*; lane 3: *hld*

The results of PCR among methicillin-resistant isolates are shown in Figure 2 and Figure 3, respectively. As shown in Table 7, the frequency of the *sea*, *seb*, *sed*, *tst*, *eta*, *etb*, *LuKS/F-PV*, *hla* and *hld* genes were 27.39%, 10.95%, 2.05%, 11.64%, 0.68%, 2.05%, 5.47%, 93.15% and 84.24%, respectively. The highest and lowest frequencies among these genes belonged to *hla* and *eta* genes, respectively. The results showed that the separate frequency of each gene in MRSA isolates from all the cases of Namazi and Faghihi hospitals indicated a significant difference, with P < 0.05.

**Table 7.** Prevalence of Various Genes of MRSA Isolated From Different Specimens

Source	*sea*	*seb*	*sed*	*tst*	*eta*	*etb*	*LukS /F-PV*	*hla*	*hld*
**Sputum**	13	3	-	3	-	-	-	56	50
**Wound**	4	6	1	5	-	1	2	17	16
**Blood**	5	1	-	4	1	1	3	19	18
**Nose**	3	-	-	1	-	1	-	3	3
**Eye**	-	-	-	-	-	-	-	3	1
**Throat**	1	-	-	-	-	-	-	4	2
**Urine**	5	-	-	-	-	-	-	11	13
**Skin**	3	4	2	4	-	-	3	8	5
**Body fluid**	-	-	-	-	-	-	-	3	3
**Endotracheal tube**	4	1	-	-	-	-	-	5	4
**Other**	2	1	-	-	-	-	-	7	8
**Total, %**	27.39	10.95	2.05	11.64	0.68	2.05	5.47	93.15	84.24

### 4.3. The Prevalence of Coexistence of Different Genes in MRSA Isolates From Various Specimens

In this study, coexistence of different genes including *sea*, *seb*, *sed*, *tst*, *eta*, *etb*, *LuKS/F-PV*, *hla* and *hld* in various specimens of MRSA was investigated. The results of the coexistence prevalence of genes are shown in Table 8. The highest coexistence of different genes belonged to *hla* + *hld* genes (48.83%) and the lowest frequency of gene coexistence were related to *hla* + *tst*, *hla* + *hld* + *LuKS/F-PV*, *hla* + *hld* + *sed*, *hla* + *seb* + *LuKS/F-PV*, *hla* + *sed* + *LuKS/F-PV*, *hla* + *hld* + *sea* + *etb*, *hla* + *hld* + *seb* + *LuKS/F-PV*, *hla* + *hld* + *sea* + *LuKS/F-PV*, *hla* + *hld* + *sed* + *sea*, *hla* + *sea* + *seb* + *tst* and *tst* + *seb* + *sea* + *LuKS/F-PV*; where each combination was detected in a single sample (0.68%). Among the 146 isolates, the endotracheal tube and body fluid specimens were devoid of any gene. As indicated in our results, 98.63% of the isolates were positive for at least one of the genes.

**Table 8.** Distribution of Toxin Genes Among Methicillin-Resistant *S. aureus* Isolated From Different Specimens ^a^

Specimens	ET (n = 6) ^b^	S (n = 58)	W (n = 18)	B (n = 20)	N (n = 3)	E (n = 3)	T (n = 4)	U (n = 14)	Sk (n = 8)	BF (n = 4)	O (n = 8)
***Hla***	-	4	-	1	-	2	2	1	-	-	-
***hld***	-	1	-	1	-	-	-	1	-	-	1
***hla*** ** + ** ***hld***	1	35	6	7	-	1	1	7	-	3	5
***hla + sea***	1	2	-	-	-	-	-	-	-	-	-
***hla + tst***	-	1	-	-	-	-	-	-	-	-	-
***hld + sea***	-	1	-	-	-	-	-	2	-	-	-
***hla + hld + sea***	2	9	1	3	1	-	1	3	-	-	1
***hla + hld + seb***	-	2	2	-	-	-	-	-	-	-	-
***hla + hld + tst***	-	2	2	2	-	-	-	-	-	-	-
***hla + hld + LukS/F-PV***	-	-	-	1	-	-	-	-	-	-	-
***hla + hld + eta***	-	-	-	1	-	-	-	-	-	-	-
***hla + hld + etb***	-	-	1	1	-	-	-	-	-	-	-
***hla + hld + sed***	-	-	1	-	-	-	-	-	-	-	-
***hla + seb +LukS/F-PV***	-	-	-	-	-	-	-	-	1	-	-
***hla + sed + LukS/F-PV***	-	-	-	-	-	-	-	-	1	-	-
***hla + hld + sea + etb***	-	-	-	-	1	-	-	-	-	-	-
***hla + hld + seb + LukS/F-PV***	-	-	1	-	-	-	-	-	-	-	-
***hla + hld + sea + LukS/F-PV***	-	-	-	1	-	-	-	-	-	-	-
***hla + hld + tst +LukS/F-PV***	-	-	-	1	-	-	-	-	1	-	-
***hla + hld + sea + seb***	1	-	-	-	-	-	-	-	-	-	1
***hla + hld + sed + sea***	-	-	-	-	-	-	-	-	1	-	-
***hla + hld + sea + tst***	-	-	1	-	1	-	-	-	1	-	-
***hla + sea + seb + tst***	-	-	-	1	-	-	-	-	-	-	-
***tst + seb + sea + LukS/F-PV***	-	-	1	-	-	-	-	-	-	-	-
***hla + sea + seb***	-	1	1	-	-	-	-	-	1	-	-
***hla + hld + seb + tst***	-	-	1	-	-	-	-	-	2	-	-

^a^ Abbreviations: B, blood; BF, body fluid; E, Eye; ET, endotracheal tube; N, nose; O, other; Sk, skin; S, sputum; T, throat; U, Urine; W, Wound.

^b^ Among ET and BF specimens only in one specimen no toxin genes were present.

## 5. Discussion

MRSA outbreaks are estimated in about 40-60% of *S. aureus* outbreaks, which are mainly affected by the infection control program and medical treatments leading to a wide range of hospital infections (21). MRSA is the cause of many nosocomial infections in Shiraz. Our study showed that the prevalence of MRSA among *S. aureus* isolates was 42.3%, which indicates little difference in terms of frequency with studies by Fatholahzadeh et al., who reported MRSA prevalence of 36% in Tehran (21). However, MRSA prevalence varies widely between different countries and may reflect the fact that different policies for infection control and other factors are involved in these areas (22). MRSA isolates were identified based on the *mecA* gene and antibiotic sensitivity tests.

This study demonstrated that cefoxitin had a sensitivity of 100% and a specificity of 99% for detecting MRSA isolates but oxacillin had 78% sensitivity and 99% specificity for detection of MRSA isolates (23). Based on the results of many studies, performed to identify MRSA and MSSA isolates, the fact that cefoxitin antibiotic is better than oxacillin antibiotic has been indicated (23-26).

The results showed that prevalence of methicillin-resistant *S. aureus* and methicillin-sensitive *S. aureus* isolates during 2012 to 2013 in Namazi and Faghihi Hospitals Shiraz were 42.3% and 57.7%, respectively. Similarly, in 2011 Jimenez et al. studied virulence genes of MSSA and MRSA strains isolated from Children's Hospital, University of Medellin, Colombia. The results of this study showed that the virulence genes in MRSA as compared to MSSA isolates had an increased diversity and repetition (83% vs. 73%) (27). *S. aureus* clinical infection is influenced by the presence of antimicrobial resistance and virulence factors. Acquisition of antibiotic resistance in *S. aureus*, including changes in the secretion of virulence factor expression and resistance in order to survive with reduced expression poison, has been suggested (7). Therefore, in this study we confirmed that diversity and abundance were greater in pathogen carriers of MSSA as compared to MRSA strains. In our study, among the 146 confirmed methicillin-resistant isolates, 54 isolates (36.98%) were females and 92 isolates (63.02%) males. In a study, Rahman et al. in Pakistan reported that among the 929 MRSA isolates, 538 were males and 391 females, respectively (28). Their results are in line with our results regarding the number of isolates from males in comparison to females. Among the 146 samples of MRSA isolated in this study, the highest proportion belonged to sputum samples with a rate of 39.79% (58 patients) whereas eye and nose samples had very low rates of only 2.05% (3 patients). Tanaka et al. reported that among the total specimens collected during 1993-1997 in Japan, most cases of MRSA (34.3%) were obtained from sputum and the least number of specimens were gathered from stool (1.8%) (29). Anagaw et al. in Ethiopia reported that among wound specimens (59.18%), MRSA had the highest isolation frequency (30). According to our studies and various re*sea*rches from other countries, it can be safely said that the most common MRSA specimens are often associated with sputum and pus. These results directly suggest that MRSA strains are responsible for most respiratory and blood infections (septicemia, endocarditis, etc.) in hospitalized patients (31-33). Also the greater frequency of respiratory specimens indicates more transmission through respiratory tract infections and nosocomial MRSA, as shown by several studies (34, 35). Our results confirm different frequencies of MRSA in various specimens. Many studies have reported that staphylococcal toxins produced are different considering the existence of different genotypes. In addition, it has been suggested that the toxin profiles of *S. aureus* in specific isolates may be affected by the origins of their geographical location (36, 37).

Several studies have been performed on toxic genes of MRSA to various dimensions, around the world. The frequency of the *sea* gene of MRSA was 58.8% in Gorgan of Iran (38), 33% China (39), 15.78% Canada, (19), 74.4% Tehran of Iran (40), 12% Germany (41), 17.5% Malaysia, (13), 32.07% Tehran of Iran (42), 27% Korea (43), 12% Czech (44) and 30% Turkey (45). In a study carried out in Colombia, among the 30 MRSA isolates, the nil gene of *sea* was not reported (27). In a study performed in the United States, the frequency of the *sea* gene was reported in the range of 54-95% (46). The frequency of the *sea* gene in MRSA isolates from various specimens obtained in our study was 27.39%, which is similar to that of the study performed in Korea (43). Moreover, the frequency of the *seb* gene has been investigated by many studies. Frequency of this gene in blood specimens in Gorgan of Iran (38), China (39), Canada (19), Tehran (42), Korea (43), Czech (44), Colombia (26) were 61.3%, 5%, 15.78%, 73.58%, 5.6%, 3%, and 7%, respectively. In our study, the frequency of the *seb* gene was 10.95%. This indicates that different frequencies in Iran in 2012 are somewhat close to the results of related studies from Canada (19).

The frequency rates of *sed* gene in blood specimen studies performed in Tehran (42), Korea (43) and Germany in 1998 (41) and Columbia in 2011 (26) were 3.77%, 2.9%, 2%, and 7%, respectively. The frequency of the *sed* gene in our study was 2.05%. Our results are consistent with those of previous studies and have many similarities. In a study from the United States, 0-13% range for the *sed* gene frequency was reported (46). The frequency of the *tst* gene in various blood and nose specimen studies performed in Germany (41), Tehran (42), Korea (43), Czech (44) and Colombia (24) were 14%, 26.41%, 72.2%, 50% and 2% , respectively. In a study from the United States, 0-78% range for the *tst* gene frequency was reported (46). The frequency of the *tst* gene in our study was 11.64%.

The frequency rates of *eta* gene reported from Germany (41), Czech (44), Turkey, (45), and Colombia (26) were 2%, 10%, 19.2%, and 3%, respectively. In a study from the United States, 0-56% range was reported for the *eta* gene frequency (46). The frequency of the *eta* gene in our study was 0.68%. The frequency of the *etb* gene in studies carried out in Turkey was 9.2% (44). In Colombia, among 30 MRSA, no *etb* gene was detected (26). Moreover, in a study in the United States, 0-22% range was reported for the *etb* gene frequency (46). However, the frequency of the *etb* in our study was 2.05%.

The frequency of *LuKS/F-PV* reported by studies performed in Ahvaz was 7.23% (47), Tehran 24.2% (42), Tehran 19% (39) Isfahan 75% (48) and Colombia 73% (11). In the Czech and Korea, of the specimens studied in 2009 no cases revealed *LuKS/F-PV* gene of MRSA (43, 44) . In a study from the United States, 0-100% range for the *LuKS/F-PV* gene frequency was reported (46). The frequency of the *LuKS/F-PV* gene in our study was 5.47 %. The frequency obtained in our study is similar to that of a study carried out in Ahvaz. This similarity could be due to geographical proximity. The frequency of *hla* gene, investigated by Kateete et al. in Uganda was reported to be 100% (49). Likewise, in a study from the United States the *hla* gene frequency was reported at 100% (46). In our study, the *hla* gene with a frequency of 93.15% was the most abundant one. According to studies conducted in other parts of the world, we can conclude that comparatively this gene is much more frequent in MRSA isolates.

Kateete et al. reported that the frequency of *hld* gene was 100% as compared to other genes in their study (49). Similarly, in a study in the United States, 100% was reported for the *hld* gene frequency. In our study the frequency of *hld* gene was 84.24%. It can thus be suggested that the *hld* gene after *hla* gene is of the highest frequency. In addition, the frequency of these genes is greater as compared to other genes in MRSA. Although genes encoding toxins are located on chromosome hemolysin, the prevalence of acquired genetic elements carrying genes can be reduced. Also in the genes encoding toxins located on chromosome hemolysin, the prevalence of acquired genetic elements carrying genes can be reduced and eventually in the genes encoding hemolysin toxins located on chromosome hemolysin, the prevalence of acquired genetic elements carrying genes can be minimized.

Other studies performed on MRSA genes suggested coexistence and other genes in the bacteria. Based on the findings of our study, the prevalence of coexisting genes in 98.63% of the isolates was positive for at least one of the genes. The existence of at least one gene of the studied genes coexisting on the MRSA isolates was 97.2% and 85.5% in the studies carried out by Emaneini et al. (40) and Kim et al. (13), respectively. The results of our study showed that most coexistent genes in the isolates were *hla* + *hld* genes with the frequency of 48.83% (66 specimens). Interestingly, in separate gene frequency analysis, each of hla or hld genes alone were revealed to be the most frequent ones. Coexistence of genes *hla* + *hld* + *sea* in combination was found in 14.58% of isolates. Coexistence of enterotoxin genes as compared to other genes encoding enterotoxin gene can be related to different genetic elements such as phages (*sea*, *see*), plasmids (*sed*) and pathogenicity islands (*seb* and *sec*) (50, 51). According to other studies, it has been found that the prevalence of genes is based on their *SCCmec* classes because some genes are more common in some classes; so, it would be better to conduct the detection of *SCCmec* classes of MRSA isolates, if subsequent prevalence study of the virulence genes is concerned. We prospect that *SCCmec* genes investigated in our study are probably of the *SCCmec* class type I. Thus, the percentage of prevalence of the genes in different SCCmec classes can be explained in the same manner. It is, thus, highly recommended that in future studies the specimens should be collected from specified sections of the hospital where the prevalence of toxin genes is optimal. Also, as mentioned earlier, we suggest that with the prevalence of these genes in MRSA specimens, primarily the *SCCmec* class should be determined until the results are justified. 

The MRSA genes frequency in different countries and even within a country and between different cities or hospitals in different parts of a city or a hospital can lead to differences. The difference in the prevalence of MRSA genes could be due to differences in geographical conditions of each country or region or part of the hospital where the specimens were collected. In conclusion, the higher frequency of some virulence genes in this study may reflect the emergence of isolates containing these genes in our medical centers in Shiraz.
